# Retraction: Artificial Intelligence–Based Neural Network for the Diagnosis of Diabetes: Model Development

**DOI:** 10.2196/98668

**Published:** 2026-04-28

**Authors:** JMIR Publications Editorial Office

**Affiliations:** 1 JMIR Publications Toronto, ON

The JMIR Publications Editorial Office is retracting the article:

Artificial Intelligence–Based Neural Network for the Diagnosis of Diabetes: Model Development [1]

This action is being taken due to identified concerns regarding potential manipulation of the submission process and the integrity of the peer review process.

The concerns with this publication were identified during an investigation of a series of articles with related concerns. Author Yue Liu was contacted and offered an opportunity to comment on these findings and the proposed retraction but did not respond to any attempts at communication.

## References

[1] Liu Y (2020). Artificial intelligence–based neural network for the diagnosis of diabetes: model development. JMIR Med Inform.

